# Crystal structure of cyclo­sulfamuron

**DOI:** 10.1107/S2056989015014115

**Published:** 2015-07-31

**Authors:** Gihaeng Kang, Jineun Kim, Eunjin Kwon, Tae Ho Kim

**Affiliations:** a Department of Chemistry and Research Institute of Natural Sciences, Gyeongsang National University, Jinju 660-701, Republic of Korea

**Keywords:** crystal structure, hydrogen bonding, π–π inter­actions

## Abstract

The title compound (systematic name: 1-{[2-(cyclo­propylcarbon­yl)anilino]sulfon­yl}-3-(4,6-di­meth­oxy­pyrimidin-2-yl)urea), C_17_H_19_N_5_O_6_S, is a pyrimidinyl­sulfonyl­urea herbicide. The dihedral angles between the mean planes of the central benzene ring and the cyclo­propyl and pyrimidinyl rings are 75.32 (9) and 88.79 (4)°, respectively. The C atoms of the meth­oxy groups lie almost in the plane of the pyrimidine ring [deviations = 0.043 (2) and 0.028 (2) Å] and intra­molecular N—H⋯N, N—H⋯O and C—H⋯O hydrogen bonds all close *S*(6) rings. In the crystal, N—H⋯O and C—H⋯O hydrogen bonds and weak π–π inter­actions [centroid–centroid distances = 3.6175 (9) and 3.7068 (9) Å] link adjacent mol­ecules, forming a three-dimensional network.

## Related literature   

For information on the herbicidal properties of the title compound, see: Sarıgül & İnam (2009 ▸). For a related crystal structure, see: Xia *et al.* (2008 ▸).
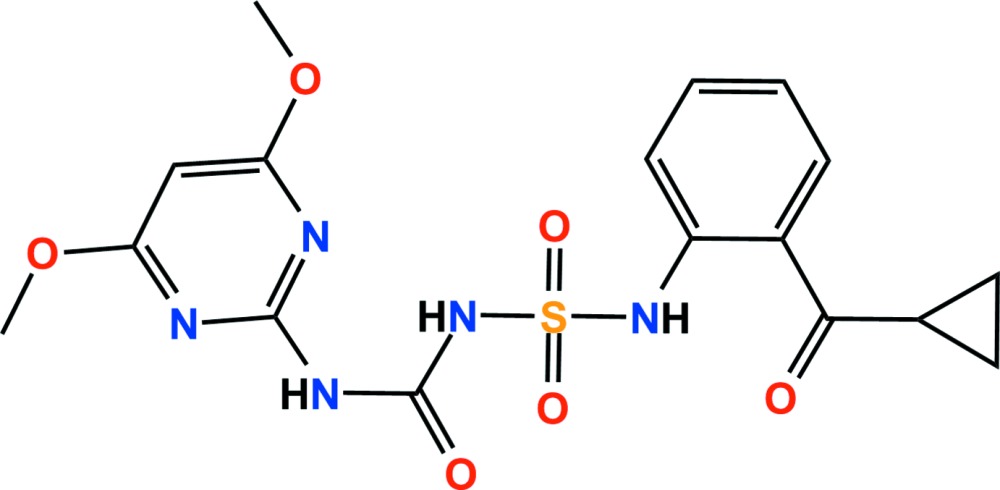


## Experimental   

### Crystal data  

C_17_H_19_N_5_O_6_S
*M* *_r_* = 421.43
Monoclinic, 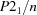

*a* = 12.7019 (4) Å
*b* = 9.6216 (3) Å
*c* = 15.6213 (5) Å
β = 93.6194 (12)°
*V* = 1905.31 (10) Å^3^
*Z* = 4
Mo *K*α radiation
μ = 0.22 mm^−1^
*T* = 173 K
0.32 × 0.27 × 0.23 mm

### Data collection  

Bruker APEXII CCD diffractometer
Absorption correction: multi-scan (*SADABS*; Bruker, 2013 ▸) *T*_min_ = 0.934, *T*_max_ = 0.952
17544 measured reflections
4365 independent reflections
3688 reflections with *I* > 2σ(*I*)
*R*_int_ = 0.030

### Refinement  

*R*[*F* ^2^ > 2σ(*F* ^2^)] = 0.039
*wR*(*F* ^2^) = 0.113
*S* = 1.05
4365 reflections
264 parameters
H-atom parameters constrained
Δρ_max_ = 0.22 e Å^−3^
Δρ_min_ = −0.49 e Å^−3^

### 

Data collection: *APEX2* (Bruker, 2013 ▸); cell refinement: *SAINT* (Bruker, 2013 ▸); data reduction: *SAINT*; program(s) used to solve structure: *SHELXS97* (Sheldrick, 2008 ▸); program(s) used to refine structure: *SHELXL2013* (Sheldrick, 2015 ▸); molecular graphics: *DIAMOND* (Brandenburg, 2010 ▸); software used to prepare material for publication: *SHELXTL* (Sheldrick, 2008 ▸).

## Supplementary Material

Crystal structure: contains datablock(s) global, I. DOI: 10.1107/S2056989015014115/hb7470sup1.cif

Structure factors: contains datablock(s) I. DOI: 10.1107/S2056989015014115/hb7470Isup2.hkl

Supporting information file. DOI: 10.1107/S2056989015014115/hb7470Isup3.cml

**Fig. 1.**
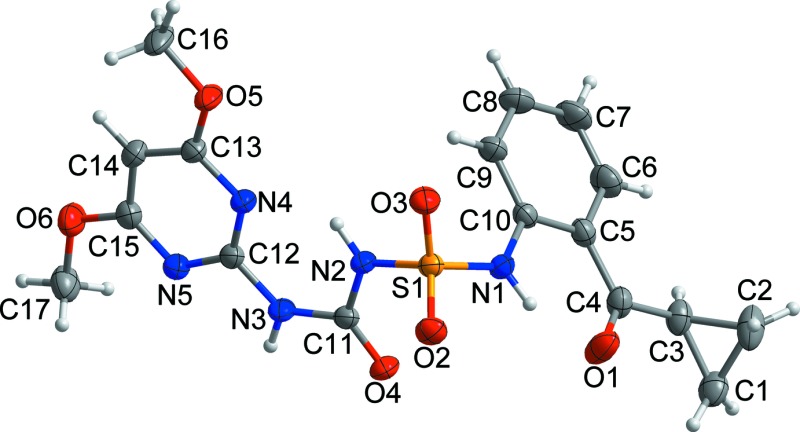
The asymmetric unit of the title compound with the atom numbering scheme. Displacement ellipsoids are drawn at the 50% probability level. H atoms are shown as small spheres of arbitrary radius.

**Fig. 2.**
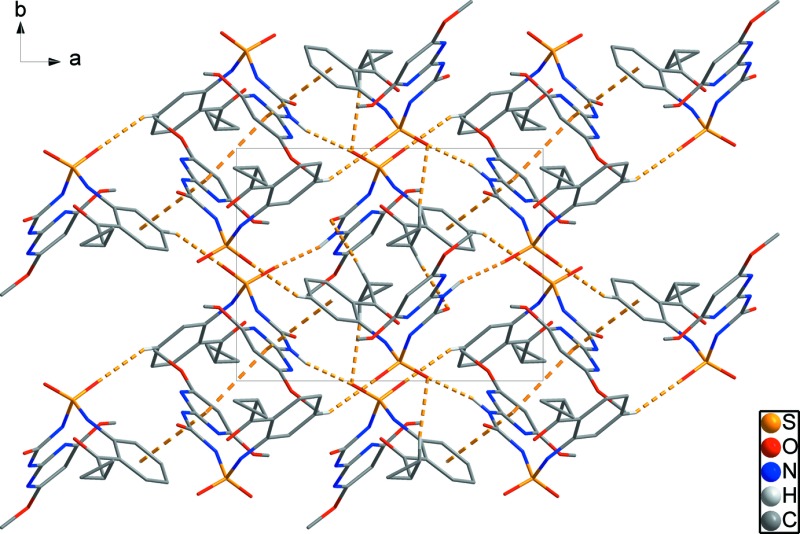
Crystal packing viewed along the c axis. The intermolecular interactions are shown as dashed lines.

. DOI: 10.1107/S2056989015014115/hb7470fig1.tif
The asymmetric unit of the title compound with the atom numbering scheme. Displacement ellipsoids are drawn at the 50% probability level. H atoms are shown as small spheres of arbitrary radius.

c . DOI: 10.1107/S2056989015014115/hb7470fig2.tif
Crystal packing viewed along the *c* axis. The inter­molecular inter­actions are shown as dashed lines.

CCDC reference: 1415211

Additional supporting information:  crystallographic information; 3D view; checkCIF report

## Figures and Tables

**Table 1:** Hydrogen-bond geometry (, )

*D*H*A*	*D*H	H*A*	*D* *A*	*D*H*A*
N1H1*N*O1	0.88	1.86	2.5736(18)	137
N2H2*N*N4	0.88	1.92	2.6158(18)	135
C9H9O3	0.95	2.45	3.088(2)	124
N3H3*N*O2^i^	0.88	2.08	2.9391(17)	165
C2H2*B*O2^ii^	0.99	2.51	3.483(2)	169
C3H3O4^iii^	1.00	2.51	3.286(2)	135
C8H8O3^iv^	0.95	2.50	3.307(2)	142

Symmetry codes: (i) 
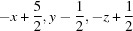
; (ii) 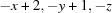; (iii) 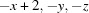; (iv) 
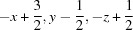
.

## supplementary crystallographic information

### S1. Comment

Cyclosulfamuron [systematic name: 1-[2-(cyclopropylcarbonyl)anilinosulfonyl]-3-(4,6-dimethoxypyrimidin-2-yl)urea] is a pyrimidinylsulfonylurea herbicide and has been widely used to control weeds because of their low toxicity to mammals and their high herbicidal activity (Sarıgül & Inam, 2009). However, until now its crystal structure has not been reported. In the title compound (Fig. 1), the dihedral angles between the mean planes of the central phenyl ring and the cyclopropyl and pyrimidinyl rings are 75.32 (9) and 88.79 (4)°, respectively. All bond lengths and bond angles are normal and comparable to those observed in a similar crystal structure (Xia *et al.*, 2008).

In the crystal structure (Fig. 2), N—H···O and C—H···O hydrogen bonds are observed (Table 1). In addition, weak intermolecular *Cg*1···*Cg*1^v^ and *Cg*1···*Cg*2^vi^ (*Cg*1 and *Cg*2 are the centroids of the N4—C12—N5—C15—C14—C13 and C5–C10 rings, respectively) interactions are present [for symmetry codes: (v), -*x* + 2, -*y*, -*z* + 1, (vi), *x* + 1/2, -*y* + 1/2, *z* + 1/2]. A three-dimensional network is formed by the hydrogen bonds and π–π interactions.

### S2. Experimental

The title compound was purchased from the Dr. Ehrenstorfer GmbH Company. Slow evaporation of a solution in CH_2_Cl_2_ gave single crystals suitable for X-ray analysis.

### S3. Refinement

All H-atoms were positioned geometrically and refined using a riding model with d(N—H) = 0.88 Å, *U*_iso_ = 1.2*U*_eq_(C) for N—H group, d(C—H) = 1.00 Å, *U*_iso_ = 1.2*U*_eq_(C) for C*sp*^3^—H. d(C—H) = 0.98 Å, *U*_iso_ = 1.5*U*_eq_(C) for methyl group, d(C—H) = 0.99 Å, *U*_iso_ = 1.2*U*_eq_(C) for CH_2_ group, d(C—H) = 0.95 Å, *U*_iso_ = 1.2*U*_eq_(C) for aromatic C—H.

### Crystal data

**Table:**

C_17_H_19_N_5_O_6_S	*F*(000) = 880
*M**_r_* = 421.43	*D*_x_ = 1.469 Mg m^−^^3^
Monoclinic, *P*2_1_/*n*	Mo *K*α radiation, λ = 0.71073 Å
*a* = 12.7019 (4) Å	Cell parameters from 6977 reflections
*b* = 9.6216 (3) Å	θ = 2.6–27.4°
*c* = 15.6213 (5) Å	µ = 0.22 mm^−^^1^
β = 93.6194 (12)°	*T* = 173 K
*V* = 1905.31 (10) Å^3^	Block, colourless
*Z* = 4	0.32 × 0.27 × 0.23 mm

### Data collection

**Table:**

Bruker APEXII CCD diffractometer	3688 reflections with *I* > 2σ(*I*)
φ and ω scans	*R*_int_ = 0.030
Absorption correction: multi-scan (*SADABS*; Bruker, 2013)	θ_max_ = 27.5°, θ_min_ = 2.0°
*T*_min_ = 0.934, *T*_max_ = 0.952	*h* = −16→15
17544 measured reflections	*k* = −12→12
4365 independent reflections	*l* = −20→20

### Refinement

**Table:**

Refinement on *F*^2^	0 restraints
Least-squares matrix: full	Hydrogen site location: inferred from neighbouring sites
*R*[*F*^2^ > 2σ(*F*^2^)] = 0.039	H-atom parameters constrained
*wR*(*F*^2^) = 0.113	*w* = 1/[σ^2^(*F*_o_^2^) + (0.0579*P*)^2^ + 0.7248*P*] where *P* = (*F*_o_^2^ + 2*F*_c_^2^)/3
*S* = 1.05	(Δ/σ)_max_ = 0.001
4365 reflections	Δρ_max_ = 0.22 e Å^−^^3^
264 parameters	Δρ_min_ = −0.49 e Å^−^^3^

### Special details

**Table:**

Geometry. All e.s.d.'s (except the e.s.d. in the dihedral angle between two l.s. planes) are estimated using the full covariance matrix. The cell e.s.d.'s are taken into account individually in the estimation of e.s.d.'s in distances, angles and torsion angles; correlations between e.s.d.'s in cell parameters are only used when they are defined by crystal symmetry. An approximate (isotropic) treatment of cell e.s.d.'s is used for estimating e.s.d.'s involving l.s. planes.

### Fractional atomic coordinates and isotropic or equivalent isotropic displacement parameters (Å^2^)

**Table:**

	*x*	*y*	*z*	*U*_iso_*/*U*_eq_	
S1	1.03274 (3)	0.41778 (4)	0.19915 (2)	0.02564 (12)	
O1	1.02757 (11)	0.25129 (16)	−0.03440 (8)	0.0524 (4)	
O2	1.12410 (9)	0.48608 (12)	0.17159 (7)	0.0340 (3)	
O3	0.95254 (9)	0.49585 (12)	0.23699 (7)	0.0343 (3)	
O4	1.19071 (9)	0.18921 (12)	0.19997 (7)	0.0335 (3)	
O5	0.94327 (9)	0.31556 (13)	0.52112 (7)	0.0386 (3)	
O6	1.17908 (10)	−0.04334 (14)	0.61128 (7)	0.0429 (3)	
N1	0.98567 (10)	0.33405 (14)	0.11638 (8)	0.0290 (3)	
H1N	1.0187	0.3456	0.0690	0.035*	
N2	1.06665 (10)	0.30293 (14)	0.27345 (8)	0.0281 (3)	
H2N	1.0317	0.3014	0.3203	0.034*	
N3	1.17533 (10)	0.13526 (15)	0.33969 (8)	0.0303 (3)	
H3N	1.2279	0.0767	0.3351	0.036*	
N4	1.05707 (10)	0.22678 (14)	0.43352 (8)	0.0284 (3)	
N5	1.17993 (10)	0.04736 (14)	0.47520 (8)	0.0302 (3)	
C1	1.00581 (16)	0.0853 (2)	−0.18258 (11)	0.0418 (4)	
H1A	1.0695	0.1442	−0.1740	0.050*	
H1B	1.0162	−0.0019	−0.2147	0.050*	
C2	0.90252 (18)	0.1560 (2)	−0.19567 (11)	0.0492 (5)	
H2A	0.8486	0.1127	−0.2357	0.059*	
H2B	0.9019	0.2589	−0.1950	0.059*	
C3	0.93094 (14)	0.08205 (17)	−0.11251 (10)	0.0354 (4)	
H3	0.8943	−0.0082	−0.1031	0.042*	
C4	0.95231 (14)	0.17112 (17)	−0.03618 (10)	0.0334 (4)	
C5	0.88087 (12)	0.16383 (17)	0.03524 (10)	0.0301 (3)	
C6	0.79319 (14)	0.0755 (2)	0.02989 (12)	0.0415 (4)	
H6	0.7797	0.0215	−0.0206	0.050*	
C7	0.72575 (15)	0.0639 (2)	0.09523 (13)	0.0462 (5)	
H7	0.6672	0.0023	0.0901	0.055*	
C8	0.74454 (14)	0.1432 (2)	0.16818 (12)	0.0432 (4)	
H8	0.6987	0.1354	0.2137	0.052*	
C9	0.82919 (13)	0.23370 (19)	0.17609 (10)	0.0347 (4)	
H9	0.8403	0.2886	0.2264	0.042*	
C10	0.89805 (12)	0.24451 (16)	0.11062 (9)	0.0267 (3)	
C11	1.14737 (12)	0.20858 (16)	0.26595 (9)	0.0267 (3)	
C12	1.13446 (12)	0.13831 (16)	0.42007 (9)	0.0272 (3)	
C13	1.01967 (12)	0.22237 (17)	0.51212 (9)	0.0294 (3)	
C14	1.05796 (13)	0.13144 (18)	0.57482 (9)	0.0325 (4)	
H14	1.0303	0.1274	0.6299	0.039*	
C15	1.13996 (13)	0.04587 (18)	0.55188 (10)	0.0314 (3)	
C16	0.89737 (16)	0.3201 (2)	0.60284 (11)	0.0514 (5)	
H16A	0.8605	0.2326	0.6124	0.077*	
H16B	0.8472	0.3974	0.6035	0.077*	
H16C	0.9531	0.3336	0.6484	0.077*	
C17	1.26553 (16)	−0.1300 (2)	0.58797 (12)	0.0463 (5)	
H17A	1.2435	−0.1851	0.5372	0.069*	
H17B	1.2862	−0.1925	0.6357	0.069*	
H17C	1.3256	−0.0714	0.5752	0.069*	

### Atomic displacement parameters (Å^2^)

**Table:**

	*U*^11^	*U*^22^	*U*^33^	*U*^12^	*U*^13^	*U*^23^
S1	0.0234 (2)	0.0291 (2)	0.02454 (19)	−0.00228 (14)	0.00254 (14)	−0.00382 (13)
O1	0.0535 (9)	0.0729 (10)	0.0323 (6)	−0.0295 (7)	0.0133 (6)	−0.0169 (6)
O2	0.0313 (6)	0.0348 (6)	0.0362 (6)	−0.0109 (5)	0.0052 (5)	−0.0047 (5)
O3	0.0321 (6)	0.0368 (6)	0.0343 (6)	0.0076 (5)	0.0039 (5)	−0.0045 (5)
O4	0.0305 (6)	0.0445 (7)	0.0263 (5)	0.0045 (5)	0.0096 (4)	−0.0049 (5)
O5	0.0368 (7)	0.0544 (8)	0.0255 (6)	0.0162 (6)	0.0090 (5)	−0.0010 (5)
O6	0.0459 (8)	0.0518 (8)	0.0309 (6)	0.0133 (6)	0.0007 (5)	0.0070 (5)
N1	0.0263 (7)	0.0387 (7)	0.0222 (6)	−0.0083 (6)	0.0039 (5)	−0.0029 (5)
N2	0.0243 (7)	0.0385 (7)	0.0221 (6)	0.0048 (5)	0.0055 (5)	−0.0010 (5)
N3	0.0256 (7)	0.0394 (7)	0.0263 (6)	0.0096 (6)	0.0059 (5)	−0.0015 (5)
N4	0.0253 (7)	0.0370 (7)	0.0232 (6)	0.0037 (5)	0.0039 (5)	−0.0023 (5)
N5	0.0263 (7)	0.0368 (7)	0.0274 (6)	0.0026 (6)	0.0016 (5)	−0.0017 (5)
C1	0.0512 (11)	0.0429 (10)	0.0312 (8)	0.0058 (8)	0.0010 (8)	−0.0081 (7)
C2	0.0760 (15)	0.0388 (10)	0.0310 (9)	0.0193 (10)	−0.0112 (9)	−0.0048 (7)
C3	0.0467 (10)	0.0305 (8)	0.0283 (8)	0.0018 (7)	−0.0033 (7)	−0.0030 (6)
C4	0.0368 (9)	0.0366 (9)	0.0262 (7)	−0.0024 (7)	−0.0031 (6)	−0.0015 (6)
C5	0.0270 (8)	0.0333 (8)	0.0292 (7)	−0.0029 (6)	−0.0049 (6)	0.0009 (6)
C6	0.0348 (10)	0.0436 (10)	0.0451 (10)	−0.0099 (8)	−0.0048 (8)	−0.0083 (8)
C7	0.0301 (9)	0.0511 (11)	0.0571 (11)	−0.0163 (8)	−0.0002 (8)	−0.0050 (9)
C8	0.0282 (9)	0.0552 (11)	0.0471 (10)	−0.0093 (8)	0.0086 (7)	0.0018 (9)
C9	0.0255 (8)	0.0450 (10)	0.0338 (8)	−0.0062 (7)	0.0027 (6)	−0.0025 (7)
C10	0.0220 (7)	0.0304 (8)	0.0272 (7)	−0.0022 (6)	−0.0019 (6)	0.0015 (6)
C11	0.0209 (7)	0.0342 (8)	0.0252 (7)	−0.0015 (6)	0.0030 (5)	−0.0046 (6)
C12	0.0234 (7)	0.0346 (8)	0.0238 (7)	−0.0015 (6)	0.0017 (6)	−0.0037 (6)
C13	0.0260 (8)	0.0379 (8)	0.0244 (7)	0.0015 (6)	0.0033 (6)	−0.0057 (6)
C14	0.0328 (9)	0.0429 (9)	0.0220 (7)	0.0020 (7)	0.0036 (6)	−0.0029 (6)
C15	0.0287 (8)	0.0383 (9)	0.0270 (7)	−0.0005 (7)	−0.0013 (6)	−0.0006 (6)
C16	0.0513 (12)	0.0780 (15)	0.0262 (8)	0.0277 (11)	0.0122 (8)	−0.0043 (9)
C17	0.0476 (11)	0.0523 (11)	0.0379 (9)	0.0167 (9)	−0.0058 (8)	0.0016 (8)

### Geometric parameters (Å, º)

**Table:**

S1—O2	1.4237 (11)	C2—C3	1.505 (2)
S1—O3	1.4238 (11)	C2—H2A	0.9900
S1—N1	1.6065 (12)	C2—H2B	0.9900
S1—N2	1.6402 (13)	C3—C4	1.480 (2)
O1—C4	1.227 (2)	C3—H3	1.0000
O4—C11	1.2132 (17)	C4—C5	1.484 (2)
O5—C13	1.3351 (19)	C5—C6	1.399 (2)
O5—C16	1.4372 (19)	C5—C10	1.416 (2)
O6—C15	1.3366 (19)	C6—C7	1.378 (3)
O6—C17	1.444 (2)	C6—H6	0.9500
N1—C10	1.4058 (19)	C7—C8	1.379 (3)
N1—H1N	0.8800	C7—H7	0.9500
N2—C11	1.3799 (19)	C8—C9	1.383 (2)
N2—H2N	0.8800	C8—H8	0.9500
N3—C11	1.378 (2)	C9—C10	1.391 (2)
N3—C12	1.3890 (18)	C9—H9	0.9500
N3—H3N	0.8800	C13—C14	1.379 (2)
N4—C12	1.327 (2)	C14—C15	1.392 (2)
N4—C13	1.3449 (19)	C14—H14	0.9500
N5—C15	1.3303 (19)	C16—H16A	0.9800
N5—C12	1.333 (2)	C16—H16B	0.9800
C1—C2	1.480 (3)	C16—H16C	0.9800
C1—C3	1.495 (2)	C17—H17A	0.9800
C1—H1A	0.9900	C17—H17B	0.9800
C1—H1B	0.9900	C17—H17C	0.9800
			
O2—S1—O3	120.02 (7)	C7—C6—C5	122.31 (17)
O2—S1—N1	104.90 (7)	C7—C6—H6	118.8
O3—S1—N1	111.04 (7)	C5—C6—H6	118.8
O2—S1—N2	110.02 (7)	C6—C7—C8	118.96 (17)
O3—S1—N2	102.97 (7)	C6—C7—H7	120.5
N1—S1—N2	107.42 (7)	C8—C7—H7	120.5
C13—O5—C16	116.90 (13)	C7—C8—C9	120.99 (17)
C15—O6—C17	116.82 (13)	C7—C8—H8	119.5
C10—N1—S1	127.72 (10)	C9—C8—H8	119.5
C10—N1—H1N	116.1	C8—C9—C10	120.18 (16)
S1—N1—H1N	116.1	C8—C9—H9	119.9
C11—N2—S1	123.13 (10)	C10—C9—H9	119.9
C11—N2—H2N	118.4	C9—C10—N1	122.06 (14)
S1—N2—H2N	118.4	C9—C10—C5	119.97 (14)
C11—N3—C12	130.81 (13)	N1—C10—C5	117.98 (13)
C11—N3—H3N	114.6	O4—C11—N3	121.65 (14)
C12—N3—H3N	114.6	O4—C11—N2	123.54 (14)
C12—N4—C13	115.60 (13)	N3—C11—N2	114.81 (12)
C15—N5—C12	114.47 (13)	N4—C12—N5	127.86 (14)
C2—C1—C3	60.76 (12)	N4—C12—N3	118.65 (14)
C2—C1—H1A	117.7	N5—C12—N3	113.49 (13)
C3—C1—H1A	117.7	O5—C13—N4	112.08 (13)
C2—C1—H1B	117.7	O5—C13—C14	125.27 (14)
C3—C1—H1B	117.7	N4—C13—C14	122.65 (14)
H1A—C1—H1B	114.8	C13—C14—C15	115.36 (14)
C1—C2—C3	60.10 (12)	C13—C14—H14	122.3
C1—C2—H2A	117.8	C15—C14—H14	122.3
C3—C2—H2A	117.8	N5—C15—O6	119.06 (15)
C1—C2—H2B	117.8	N5—C15—C14	124.04 (15)
C3—C2—H2B	117.8	O6—C15—C14	116.89 (14)
H2A—C2—H2B	114.9	O5—C16—H16A	109.5
C4—C3—C1	119.02 (16)	O5—C16—H16B	109.5
C4—C3—C2	116.34 (14)	H16A—C16—H16B	109.5
C1—C3—C2	59.14 (12)	O5—C16—H16C	109.5
C4—C3—H3	116.6	H16A—C16—H16C	109.5
C1—C3—H3	116.6	H16B—C16—H16C	109.5
C2—C3—H3	116.6	O6—C17—H17A	109.5
O1—C4—C3	119.04 (15)	O6—C17—H17B	109.5
O1—C4—C5	121.68 (14)	H17A—C17—H17B	109.5
C3—C4—C5	119.26 (15)	O6—C17—H17C	109.5
C6—C5—C10	117.59 (15)	H17A—C17—H17C	109.5
C6—C5—C4	120.40 (15)	H17B—C17—H17C	109.5
C10—C5—C4	122.01 (14)		
			
O2—S1—N1—C10	176.89 (13)	C4—C5—C10—C9	−179.69 (15)
O3—S1—N1—C10	−52.05 (15)	C6—C5—C10—N1	−179.72 (15)
N2—S1—N1—C10	59.84 (15)	C4—C5—C10—N1	0.3 (2)
O2—S1—N2—C11	−47.34 (14)	C12—N3—C11—O4	−177.90 (15)
O3—S1—N2—C11	−176.41 (12)	C12—N3—C11—N2	1.5 (2)
N1—S1—N2—C11	66.31 (13)	S1—N2—C11—O4	−10.3 (2)
C2—C1—C3—C4	−105.05 (18)	S1—N2—C11—N3	170.31 (11)
C1—C2—C3—C4	109.56 (18)	C13—N4—C12—N5	−0.8 (2)
C1—C3—C4—O1	4.1 (2)	C13—N4—C12—N3	178.68 (14)
C2—C3—C4—O1	−63.6 (2)	C15—N5—C12—N4	1.3 (2)
C1—C3—C4—C5	−177.36 (15)	C15—N5—C12—N3	−178.23 (14)
C2—C3—C4—C5	114.96 (19)	C11—N3—C12—N4	−2.0 (2)
O1—C4—C5—C6	177.40 (17)	C11—N3—C12—N5	177.54 (15)
C3—C4—C5—C6	−1.1 (2)	C16—O5—C13—N4	−179.99 (16)
O1—C4—C5—C10	−2.6 (3)	C16—O5—C13—C14	−0.3 (2)
C3—C4—C5—C10	178.88 (14)	C12—N4—C13—O5	179.06 (13)
C10—C5—C6—C7	−1.0 (3)	C12—N4—C13—C14	−0.6 (2)
C4—C5—C6—C7	179.00 (18)	O5—C13—C14—C15	−178.31 (15)
C5—C6—C7—C8	0.7 (3)	N4—C13—C14—C15	1.3 (2)
C6—C7—C8—C9	0.4 (3)	C12—N5—C15—O6	178.96 (14)
C7—C8—C9—C10	−1.0 (3)	C12—N5—C15—C14	−0.4 (2)
C8—C9—C10—N1	−179.31 (16)	C17—O6—C15—N5	1.8 (2)
C8—C9—C10—C5	0.7 (3)	C17—O6—C15—C14	−178.78 (15)
S1—N1—C10—C9	10.3 (2)	C13—C14—C15—N5	−0.8 (2)
S1—N1—C10—C5	−169.74 (12)	C13—C14—C15—O6	179.85 (15)
C6—C5—C10—C9	0.3 (2)		

### Hydrogen-bond geometry (Å, º)

**Table:**

*D*—H···*A*	*D*—H	H···*A*	*D*···*A*	*D*—H···*A*
N1—H1*N*···O1	0.88	1.86	2.5736 (18)	137
N2—H2*N*···N4	0.88	1.92	2.6158 (18)	135
C9—H9···O3	0.95	2.45	3.088 (2)	124
N3—H3*N*···O2^i^	0.88	2.08	2.9391 (17)	165
C2—H2*B*···O2^ii^	0.99	2.51	3.483 (2)	169
C3—H3···O4^iii^	1.00	2.51	3.286 (2)	135
C8—H8···O3^iv^	0.95	2.50	3.307 (2)	142

Symmetry codes: (i) −*x*+5/2, *y*−1/2, −*z*+1/2; (ii) −*x*+2, −*y*+1, −*z*; (iii) −*x*+2, −*y*, −*z*; (iv) −*x*+3/2, *y*−1/2, −*z*+1/2.
