# Supplementary figures and images for: Crystal structure of cyclo­sulfamuron
Source: Acta Crystallogr E Crystallogr Commun. 2015 Jul 31;71(Pt 8):o631–2. doi: 10.1107/S2056989015014115 (PMC4571433; doi:10.1107/S2056989015014115)

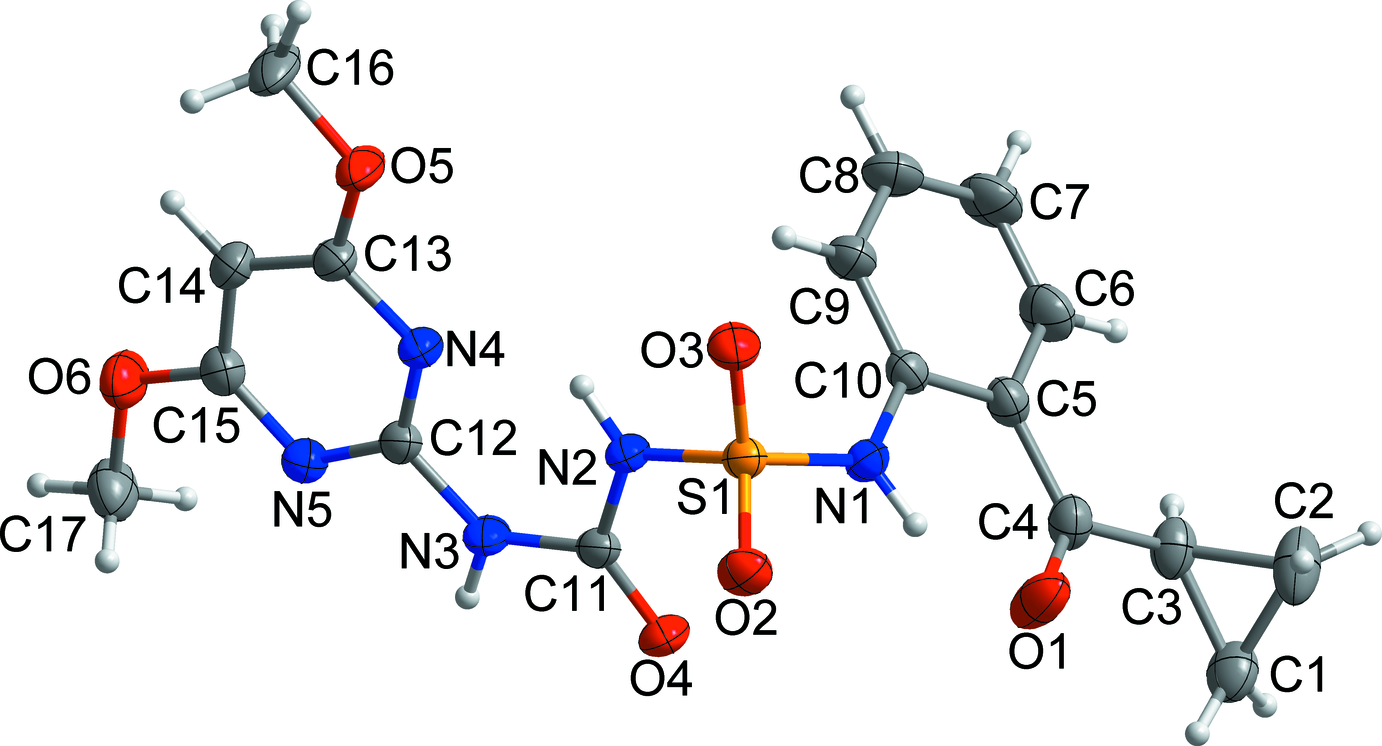

Supplement: Supplementary file 4 [file e-71-0o631-fig1.tif]

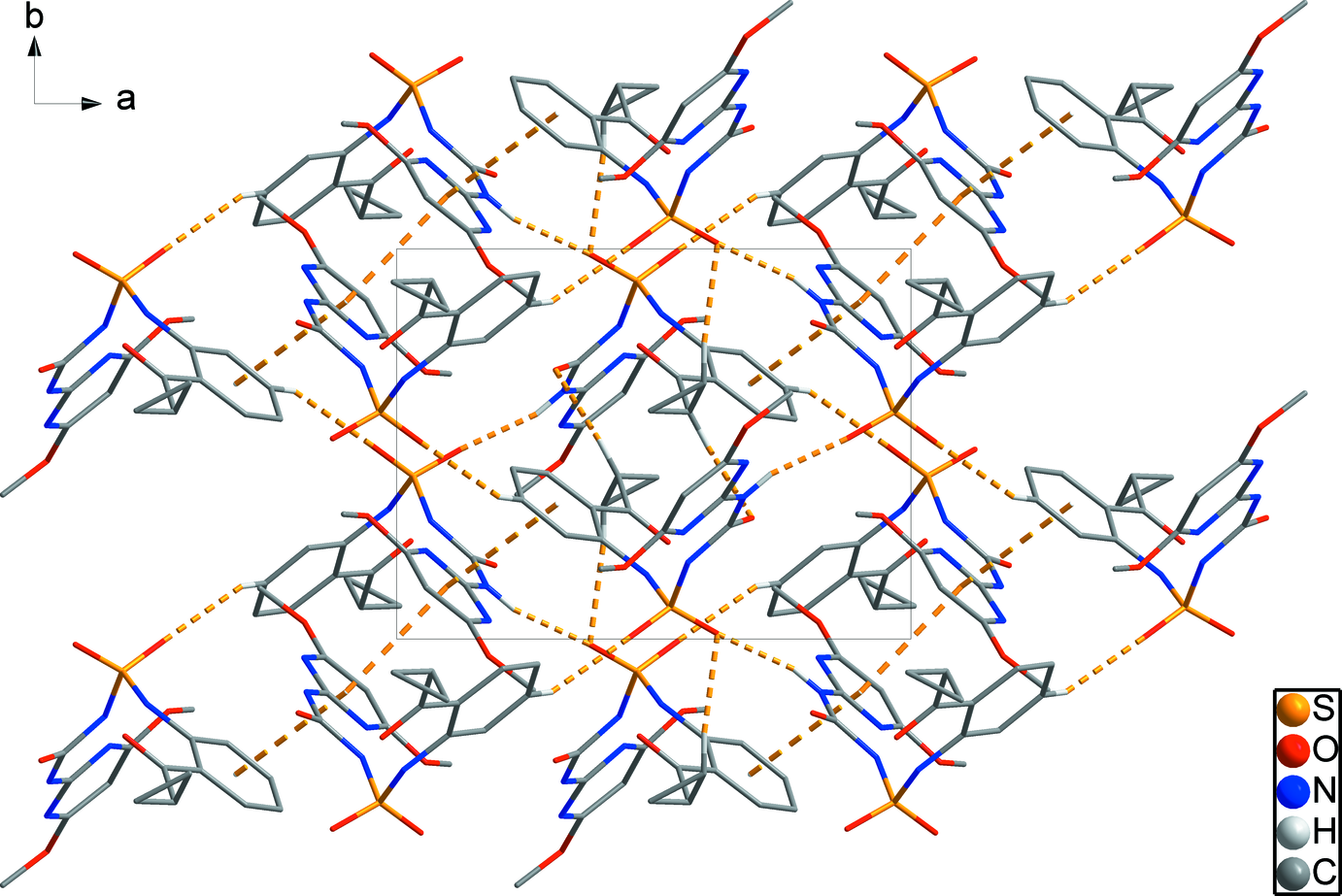

Supplement: Supplementary file 5 [file e-71-0o631-fig2.tif]
